# *meso*-4,5-Diphenyl­imidazolidin-2-one

**DOI:** 10.1107/S1600536809046133

**Published:** 2009-11-14

**Authors:** Henry Galas, Russell D. Viirre, Alan J. Lough

**Affiliations:** a Department of Chemistry and Biology, Ryerson University, Toronto, Ontario, Canada M5B 2K3; b Department of Chemistry, University of Toronto, 80 St George St, Toronto, Ontario, Canada M5B 2K3

## Abstract

The crystal structure determination of the title compound, C_15_H_14_N_2_O, confirms the *cis* relationship between the phenyl groups at the 4- and 5-positions on the imidazolidine ring. The dihedral angle between the two phenyl rings is 48.14 (6)°. In the crystal structure, inter­molecular N—H⋯O hydrogen bonds link mol­ecules into centrosymmetric dimers. These dimers are, in turn, linked into a two-dimensional network *via* weak N—H⋯π(arene) inter­actions and π–π stacking inter­actions with centroid–centroid distances of 3.6937 (11) Å.

## Related literature

For the first synthesis of this compound, see: Biniecki & Moll (1974 ▶). For the synthesis of the *trans*-isomers, see: Sankhavasi *et al.* (1991 ▶). For the crystal structure of the (*R*,*R*)-isomer, see: Siegler & Long (2006 ▶). For the synthesis of the precursor, see: Proskurnina *et al.* (2002 ▶). For applications of related enanti­opure compounds, see: Sankhavasi *et al.* (1991 ▶); Isobe *et al.* (1998 ▶); Lou *et al.* (2004 ▶). For potential applications of the title compound, see: Porosa & Viirre (2009 ▶).
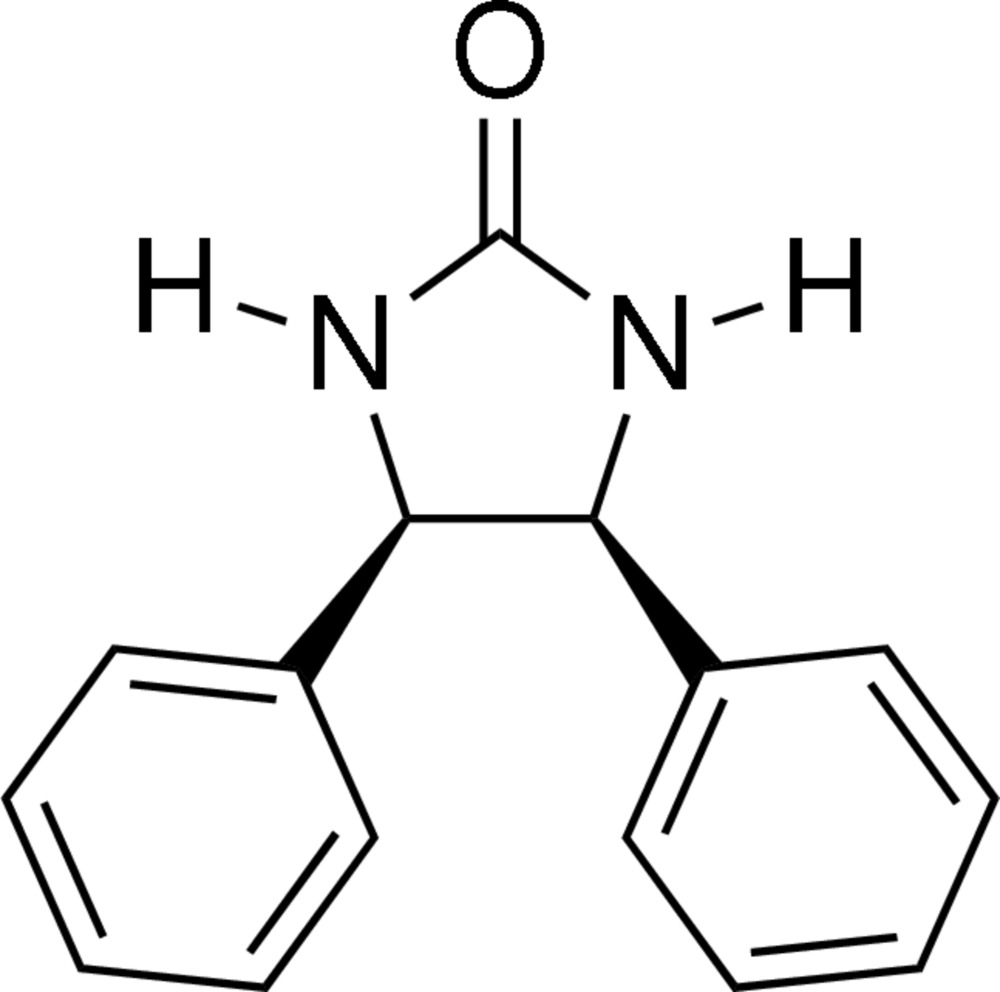


## Experimental

### 

#### Crystal data

C_15_H_14_N_2_O
*M* *_r_* = 238.28
Triclinic, 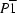

*a* = 6.3539 (4) Å
*b* = 8.6159 (4) Å
*c* = 11.3211 (7) Å
α = 86.147 (3)°
β = 76.094 (3)°
γ = 82.718 (3)°
*V* = 596.32 (6) Å^3^
*Z* = 2
Mo *K*α radiation
μ = 0.09 mm^−1^
*T* = 150 K
0.20 × 0.20 × 0.08 mm

#### Data collection

Nonius KappaCCD diffractometer
Absorption correction: multi-scan (*SORTAV*; Blessing, 1995 ▶) *T*_min_ = 0.873, *T*_max_ = 0.995
6535 measured reflections
2685 independent reflections
1771 reflections with *I* > 2σ(*I*)
*R*_int_ = 0.047

#### Refinement

*R*[*F* ^2^ > 2σ(*F* ^2^)] = 0.054
*wR*(*F* ^2^) = 0.156
*S* = 1.03
2685 reflections
172 parameters
H atoms treated by a mixture of independent and constrained refinement
Δρ_max_ = 0.25 e Å^−3^
Δρ_min_ = −0.25 e Å^−3^

### 

Data collection: *COLLECT* (Nonius, 2002 ▶); cell refinement: *DENZO-SMN* (Otwinowski & Minor, 1997 ▶); data reduction: *DENZO-SMN*; program(s) used to solve structure: *SIR92* (Altomare *et al.*, 1994 ▶); program(s) used to refine structure: *SHELXTL* (Sheldrick, 2008 ▶); molecular graphics: *PLATON* (Spek, 2009 ▶); software used to prepare material for publication: *SHELXTL*.

## Supplementary Material

Crystal structure: contains datablocks global, I. DOI: 10.1107/S1600536809046133/sj2668sup1.cif

Structure factors: contains datablocks I. DOI: 10.1107/S1600536809046133/sj2668Isup2.hkl

Additional supplementary materials:  crystallographic information; 3D view; checkCIF report

## Figures and Tables

**Table 1:** Hydrogen-bond geometry (Å, °)

*D*—H⋯*A*	*D*—H	H⋯*A*	*D*⋯*A*	*D*—H⋯*A*
N1—H1*N*⋯O1^i^	0.93 (2)	1.94 (2)	2.864 (2)	173 (2)
N2—H2*N*⋯*Cg*1^ii^	0.87 (2)	2.46 (2)	3.322 (2)	165 (2)

Symmetry codes: (i) 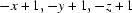; (ii) 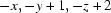. *Cg*1 is the centroid of the C4–C9 ring.

**Fig. 1.**
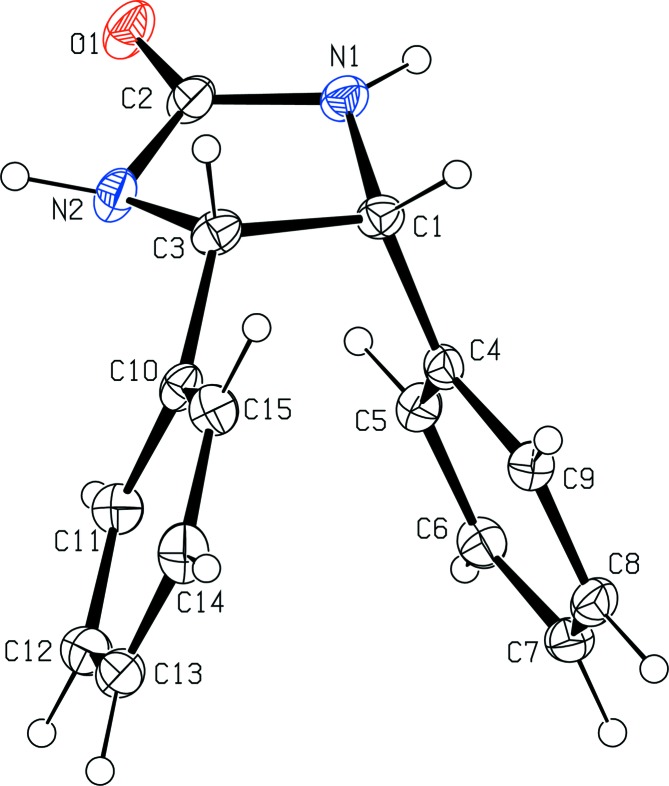
The molecular structure showing 30% probability displacement ellipsoids (arbitrary spheres for H atoms).

**Fig. 2.**
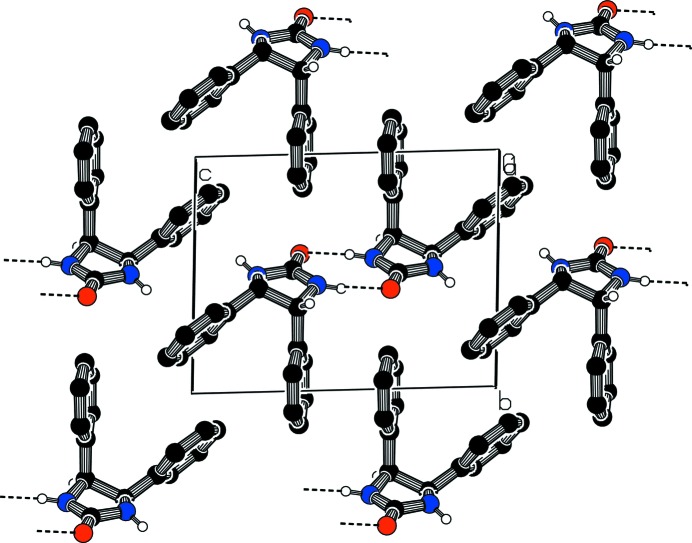
Part of the crystal structure showing hydrogen bonds as dashed lines.

**Fig. 3.**
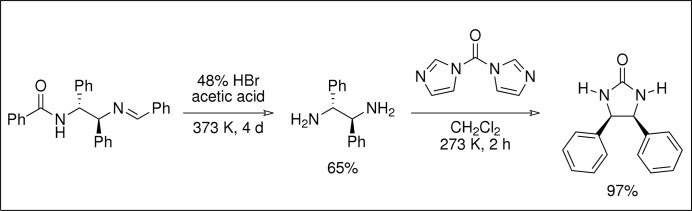
The reaction scheme.

## supplementary crystallographic information

### Comment

The title compound is a *meso*-compound, and is therefore achiral. The *trans*-isomer is chiral, and both antipodal isomers have been synthesized (Sankhavasi *et al.*, 1991). The crystal structure of the (4*R*,5*R*)-isomer has already been determined (Siegler & Long, 2006). Enantiopure samples of the *trans*-isomers have found use as precursors for chiral auxiliaries (Sankhavasi *et al.*, 1991), chiral catalysts (Isobe *et al.*, 1998), and chiral ligands (Lou *et al.*, 2004). The title compound might also be of similar use if desymmetrization can be accomplished by selective reaction of one of the two enantiotopic nitrogen atoms, for instance using an enantioselective Buchwald-Hartwig reaction (Porosa & Viirre (2009).

The title compound was prepared according to the reaction scheme shown in Fig. 3. The imine-amide precursor is readily prepared by heating benzaldehyde with NH_4_OAc according to a literature procedure (Proskurnina *et al.*, 2002). This compound was subjected to exhaustive hydrolysis, by heating in a mixture of HBr and acetic acid for four days, and the resultant *meso*-diamine was then treated with carbonyl diimidazole, resulting in the title compound.

The molecular structure is shown in Fig. 1 and confirms the *cis*-relationship between the phenyl groups at the 4 and 5 positions (atoms C1 and C3 by the crystallographic labelling scheme). This relative stereochemistry is initially set in the formation of the imine-amide species, which involves an electrocyclization governed by orbital symmetry considerations. Epimerization did not occur, even upon prolonged exposure to strong acid and heat in the hydrolysis of the imine and amide groups.

The dihedral angle between the two phenyl rings (C4—C9 and C10—C15) is 48.14 (6)°. In the crystal structure, intermolecular N—H···O hydrogen bonds link molecules into centrostmmetric dimers (Fig. 2). These dimers, are in turn, linked into a two-dimensional network *via* weak N—H···π(arene) interactions and π–π stacking interactions with *Cg*1···*Cg*1(-*x*, 2 - *y*, 1 - *z*) = 3.6937 (11) Å, where *Cg*1 is the centroid defined by ring atoms C4—C9.

### Experimental

A suspension of 1,2-diamino-*N*-benzoyl-*N*'-benzylidene-1,2-diphenylethane (23.0 g, 57 mmol) in a mixture of glacial acetic acid (115 ml) and 48% aqueous HBr (230 ml) was heated to reflux for four days. The mixture was then cooled in an ice bath and diethyl ether (200 ml) was added and vigourous stirring was continued for 30 minutes before being filtered and washed with diethyl ether. The solid filtrate was added to 100 ml of ice-cold 40% aqueous NaOH, which was then extracted three times with CH_2_Cl_2_ (150 ml). The organic extracts were evaporated to dryness and recrystallized from water to obtain *meso*-1,2-diamino-1,2-diphenylethane (7.8 g, 65% yield). ^1^H NMR [400 MHz, CDCl_3_] δH 7.41–7.26 (m, 10H), 4.02 (s, 2H), 1.33 (s, 4H). ^13^C NMR [100 MHz, CDCl_3_] δC 142.8, 128.3, 127.5, 127.4, 62.7. A portion of this diamine (5.095 g, 24 mmol) was dissolved in CH_2_Cl_2_ (50 ml) and cooled in an ice bath, while a solution of 1,1'-carbonyldiimidazole (8.108 g, 50 mmol) in CH_2_Cl_2_ (250 ml) was added dropwise. The mixture was stirred for two hours, and the solvent was evaporated under reduced pressure. The solid was taken up in MeOH (200 ml), cooled in an ice bath, 20 ml of 40% aqueous NaOH was added, and the mixture was stirred for 30 minutes. Methanol was evaporated under reduced pressure, and the remaining aqueous solution was cooled in an ice bath and acidified to pH = 1 with 0.5 *M* HCl, upon which the title compound crystallized. The crystals were filtered to obtain *meso*-4,5-diphenylimidazolin-2-one (5.560 g, 97% yield). ^1^H NMR [400 MHz, CDCl_3_] δH 7.10–7.05 (m, 6H), 6.97–6.93 (m, 4H), 5.17 (s, 2H), 5.01 (broad s, 2H). ^13^C NMR [100 MHz, CDCl_3_] δC 163.5, 137.0, 128.0, 127.8, 127.0, 61.8.

### Refinement

H atoms bound to C were placed in calculated positions with C—H distances in the range 0.95–1.00Å and included in the refinement in a riding-model approximation with *U*_iso_(H) = 1.2*U*_eq_(C). The H atoms bonded to N atoms were refined independently with isotropic displacement parameters.

### Crystal data

**Table:**

C_15_H_14_N_2_O	*Z* = 2
*M**_r_* = 238.28	*F*(000) = 252
Triclinic, *P*1	*D*_x_ = 1.327 Mg m^−^^3^
Hall symbol: -P 1	Mo *K*α radiation, λ = 0.71073 Å
*a* = 6.3539 (4) Å	Cell parameters from 6535 reflections
*b* = 8.6159 (4) Å	θ = 3.0–27.5°
*c* = 11.3211 (7) Å	µ = 0.09 mm^−^^1^
α = 86.147 (3)°	*T* = 150 K
β = 76.094 (3)°	Plate, colourless
γ = 82.718 (3)°	0.20 × 0.20 × 0.08 mm
*V* = 596.32 (6) Å^3^	

### Data collection

**Table:**

Nonius KappaCCD diffractometer	2685 independent reflections
Radiation source: fine-focus sealed tube	1771 reflections with *I* > 2σ(*I*)
graphite	*R*_int_ = 0.047
Detector resolution: 9 pixels mm^-1^	θ_max_ = 27.5°, θ_min_ = 3.0°
ω scans	*h* = −8→8
Absorption correction: multi-scan (*SORTAV*; Blessing, 1995)	*k* = −11→11
*T*_min_ = 0.873, *T*_max_ = 0.995	*l* = −12→14
6535 measured reflections	

### Refinement

**Table:**

Refinement on *F*^2^	Secondary atom site location: difference Fourier map
Least-squares matrix: full	Hydrogen site location: inferred from neighbouring sites
*R*[*F*^2^ > 2σ(*F*^2^)] = 0.054	H atoms treated by a mixture of independent and constrained refinement
*wR*(*F*^2^) = 0.156	*w* = 1/[σ^2^(*F*_o_^2^) + (0.0787*P*)^2^ + 0.1078*P*] where *P* = (*F*_o_^2^ + 2*F*_c_^2^)/3
*S* = 1.03	(Δ/σ)_max_ < 0.001
2685 reflections	Δρ_max_ = 0.25 e Å^−^^3^
172 parameters	Δρ_min_ = −0.25 e Å^−^^3^
0 restraints	Extinction correction: *SHELXTL* (Version 6.1; Sheldrick, 2008), Fc^*^=kFc[1+0.001xFc^2^λ^3^/sin(2θ)]^-1/4^
Primary atom site location: structure-invariant direct methods	Extinction coefficient: 0.043 (9)

### Special details

**Table:**

Geometry. All e.s.d.'s (except the e.s.d. in the dihedral angle between two l.s. planes) are estimated using the full covariance matrix. The cell e.s.d.'s are taken into account individually in the estimation of e.s.d.'s in distances, angles and torsion angles; correlations between e.s.d.'s in cell parameters are only used when they are defined by crystal symmetry. An approximate (isotropic) treatment of cell e.s.d.'s is used for estimating e.s.d.'s involving l.s. planes.
Refinement. Refinement of *F*^2^ against ALL reflections. The weighted *R*-factor *wR* and goodness of fit *S* are based on *F*^2^, conventional *R*-factors *R* are based on *F*, with *F* set to zero for negative *F*^2^. The threshold expression of *F*^2^ > σ(*F*^2^) is used only for calculating *R*-factors(gt) *etc*. and is not relevant to the choice of reflections for refinement. *R*-factors based on *F*^2^ are statistically about twice as large as those based on *F*, and *R*- factors based on ALL data will be even larger.

### Fractional atomic coordinates and isotropic or equivalent isotropic displacement parameters (Å^2^)

**Table:**

	*x*	*y*	*z*	*U*_iso_*/*U*_eq_	
O1	0.5277 (2)	0.41749 (16)	0.65182 (13)	0.0402 (4)	
N1	0.2254 (3)	0.55213 (18)	0.59441 (15)	0.0298 (4)	
N2	0.2105 (3)	0.50802 (19)	0.79050 (15)	0.0310 (4)	
C1	0.0274 (3)	0.6470 (2)	0.65473 (17)	0.0264 (4)	
H1A	−0.0938	0.6328	0.6156	0.032*	
C2	0.3394 (3)	0.4852 (2)	0.67556 (18)	0.0308 (5)	
C3	−0.0134 (3)	0.5630 (2)	0.78442 (17)	0.0283 (5)	
H3A	−0.0926	0.4701	0.7831	0.034*	
C4	0.0525 (3)	0.8203 (2)	0.65330 (16)	0.0243 (4)	
C5	0.2551 (3)	0.8742 (2)	0.63592 (17)	0.0288 (5)	
H5A	0.3829	0.8014	0.6267	0.035*	
C6	0.2724 (3)	1.0331 (2)	0.63194 (18)	0.0315 (5)	
H6A	0.4118	1.0687	0.6195	0.038*	
C7	0.0879 (3)	1.1399 (2)	0.64589 (18)	0.0318 (5)	
H7A	0.1004	1.2489	0.6430	0.038*	
C8	−0.1151 (3)	1.0882 (2)	0.66414 (17)	0.0317 (5)	
H8A	−0.2426	1.1614	0.6741	0.038*	
C9	−0.1316 (3)	0.9287 (2)	0.66781 (17)	0.0278 (5)	
H9A	−0.2713	0.8934	0.6805	0.033*	
C10	−0.1371 (3)	0.6618 (2)	0.88918 (16)	0.0259 (4)	
C11	−0.0311 (3)	0.7520 (2)	0.94856 (17)	0.0297 (5)	
H11A	0.1228	0.7514	0.9233	0.036*	
C12	−0.1480 (3)	0.8430 (2)	1.04446 (18)	0.0338 (5)	
H12A	−0.0735	0.9030	1.0852	0.041*	
C13	−0.3712 (3)	0.8468 (2)	1.08089 (18)	0.0339 (5)	
H13A	−0.4510	0.9103	1.1459	0.041*	
C14	−0.4787 (3)	0.7580 (2)	1.02265 (19)	0.0364 (5)	
H14A	−0.6328	0.7603	1.0479	0.044*	
C15	−0.3626 (3)	0.6652 (2)	0.92714 (17)	0.0307 (5)	
H15A	−0.4376	0.6039	0.8878	0.037*	
H2N	0.248 (3)	0.441 (3)	0.846 (2)	0.035 (6)*	
H1N	0.297 (3)	0.569 (2)	0.514 (2)	0.040 (6)*	

### Atomic displacement parameters (Å^2^)

**Table:**

	*U*^11^	*U*^22^	*U*^33^	*U*^12^	*U*^13^	*U*^23^
O1	0.0377 (9)	0.0356 (8)	0.0365 (9)	0.0126 (7)	0.0019 (7)	0.0053 (6)
N1	0.0339 (10)	0.0250 (8)	0.0261 (9)	0.0018 (7)	−0.0011 (7)	−0.0002 (7)
N2	0.0309 (10)	0.0292 (9)	0.0270 (9)	0.0048 (7)	−0.0014 (7)	0.0069 (7)
C1	0.0257 (10)	0.0249 (9)	0.0265 (10)	−0.0011 (8)	−0.0025 (8)	−0.0022 (8)
C2	0.0338 (12)	0.0231 (10)	0.0314 (11)	0.0017 (8)	−0.0030 (9)	0.0025 (8)
C3	0.0265 (10)	0.0236 (9)	0.0324 (10)	−0.0035 (8)	−0.0019 (8)	0.0000 (8)
C4	0.0262 (10)	0.0269 (10)	0.0189 (9)	−0.0010 (8)	−0.0048 (7)	0.0006 (7)
C5	0.0279 (11)	0.0279 (10)	0.0299 (10)	−0.0008 (8)	−0.0070 (8)	0.0013 (8)
C6	0.0328 (11)	0.0335 (11)	0.0296 (11)	−0.0088 (9)	−0.0077 (9)	0.0003 (8)
C7	0.0420 (12)	0.0253 (10)	0.0270 (10)	−0.0048 (9)	−0.0056 (9)	−0.0015 (8)
C8	0.0334 (12)	0.0284 (10)	0.0292 (11)	0.0047 (9)	−0.0041 (9)	0.0001 (8)
C9	0.0245 (10)	0.0302 (10)	0.0275 (10)	−0.0021 (8)	−0.0047 (8)	0.0006 (8)
C10	0.0262 (10)	0.0227 (9)	0.0259 (10)	−0.0024 (8)	−0.0026 (8)	0.0057 (7)
C11	0.0269 (10)	0.0310 (10)	0.0296 (10)	−0.0081 (8)	−0.0017 (8)	0.0013 (8)
C12	0.0444 (13)	0.0292 (10)	0.0277 (11)	−0.0119 (9)	−0.0045 (9)	0.0012 (8)
C13	0.0402 (13)	0.0303 (11)	0.0258 (10)	0.0024 (9)	−0.0009 (9)	0.0001 (8)
C14	0.0262 (11)	0.0460 (13)	0.0326 (11)	0.0013 (9)	−0.0024 (9)	0.0032 (10)
C15	0.0288 (11)	0.0336 (11)	0.0288 (10)	−0.0052 (8)	−0.0047 (8)	−0.0001 (8)

### Geometric parameters (Å, °)

**Table:**

O1—C2	1.238 (2)	C6—H6A	0.9500
N1—C2	1.358 (3)	C7—C8	1.382 (3)
N1—C1	1.456 (2)	C7—H7A	0.9500
N1—H1N	0.93 (2)	C8—C9	1.389 (3)
N2—C2	1.373 (3)	C8—H8A	0.9500
N2—C3	1.458 (3)	C9—H9A	0.9500
N2—H2N	0.87 (2)	C10—C11	1.389 (3)
C1—C4	1.520 (2)	C10—C15	1.391 (3)
C1—C3	1.571 (3)	C11—C12	1.387 (3)
C1—H1A	1.0000	C11—H11A	0.9500
C3—C10	1.506 (3)	C12—C13	1.375 (3)
C3—H3A	1.0000	C12—H12A	0.9500
C4—C9	1.386 (3)	C13—C14	1.380 (3)
C4—C5	1.389 (3)	C13—H13A	0.9500
C5—C6	1.384 (3)	C14—C15	1.392 (3)
C5—H5A	0.9500	C14—H14A	0.9500
C6—C7	1.379 (3)	C15—H15A	0.9500
			
C2—N1—C1	111.46 (16)	C5—C6—H6A	119.9
C2—N1—H1N	119.9 (13)	C6—C7—C8	119.96 (17)
C1—N1—H1N	123.4 (13)	C6—C7—H7A	120.0
C2—N2—C3	110.27 (16)	C8—C7—H7A	120.0
C2—N2—H2N	113.6 (14)	C7—C8—C9	119.57 (18)
C3—N2—H2N	124.9 (14)	C7—C8—H8A	120.2
N1—C1—C4	113.60 (15)	C9—C8—H8A	120.2
N1—C1—C3	99.73 (14)	C4—C9—C8	121.04 (17)
C4—C1—C3	114.88 (14)	C4—C9—H9A	119.5
N1—C1—H1A	109.4	C8—C9—H9A	119.5
C4—C1—H1A	109.4	C11—C10—C15	118.79 (18)
C3—C1—H1A	109.4	C11—C10—C3	121.43 (17)
O1—C2—N1	126.79 (18)	C15—C10—C3	119.77 (17)
O1—C2—N2	125.27 (18)	C12—C11—C10	120.59 (18)
N1—C2—N2	107.93 (17)	C12—C11—H11A	119.7
N2—C3—C10	113.62 (16)	C10—C11—H11A	119.7
N2—C3—C1	100.24 (14)	C13—C12—C11	120.32 (19)
C10—C3—C1	116.28 (14)	C13—C12—H12A	119.8
N2—C3—H3A	108.8	C11—C12—H12A	119.8
C10—C3—H3A	108.8	C12—C13—C14	119.72 (19)
C1—C3—H3A	108.8	C12—C13—H13A	120.1
C9—C4—C5	118.61 (16)	C14—C13—H13A	120.1
C9—C4—C1	119.26 (16)	C13—C14—C15	120.36 (19)
C5—C4—C1	122.12 (16)	C13—C14—H14A	119.8
C6—C5—C4	120.56 (18)	C15—C14—H14A	119.8
C6—C5—H5A	119.7	C10—C15—C14	120.20 (18)
C4—C5—H5A	119.7	C10—C15—H15A	119.9
C7—C6—C5	120.26 (18)	C14—C15—H15A	119.9
C7—C6—H6A	119.9		

### Hydrogen-bond geometry (Å, °)

**Table:**

*D*—H···*A*	*D*—H	H···*A*	*D*···*A*	*D*—H···*A*
N1—H1N···O1^i^	0.93 (2)	1.94 (2)	2.864 (2)	173 (2)
N2—H2N···Cg1^ii^	0.87 (2)	2.46 (2)	3.322 (2)	165 (2)

Symmetry codes: (i) −*x*+1, −*y*+1, −*z*+1; (ii) −*x*, −*y*+1, −*z*+2.
